# Rational budgeting approach as a nutrient management tool for mixed crop-swine farms in Korea

**DOI:** 10.5713/ajas.19.0640

**Published:** 2019-12-24

**Authors:** Arif Reza, Soomin Shim, Seungsoo Kim, Sungil Ahn, Seunggun Won, Changsix Ra

**Affiliations:** 1 Department of Animal Industry Convergence, College of Animal Life Science, Kangwon National University, Chuncheon 24341, Korea; 2 Department of Environmental Science, College of Agricultural Sciences, IUBAT—International University of Business Agriculture and Technology, Dhaka 1230, Bangladesh; 3 Department of Animal Resources, College of Life and Environmental Science, Daegu University, Gyeongsan 38453, Korea

**Keywords:** Nutrient Budget, Nutrient Management, Mixed Crop-swine Farm, Korea

## Abstract

**Objective:**

Due to rapid economic return, mixed crop-swine farming systems in Korea have become more intensive. Intensive farming practices often cause nutrient surpluses and lead to environmental pollution. Nutrient budgets can be used to evaluate the environmental impact and as a regulatory policy instrument for nutrient management. This study was conducted to select a nutrient budgeting approach applicable to the mixed crop-swine farms in Korea and suggest an effective manure treatment method to reduce on-farm nutrient production.

**Methods:**

In this study, we compared current and ideal gross nutrient balance (GNB) approaches of Organisation for Economic Co-operation and Development and soil system budget (SSB) approach with reference to on-farm manure treatment processes. Data obtained from farm census and published literature were used to develop the farm nutrient budgets.

**Results:**

The average nitrogen (N) and phosphorus (P) surpluses were approximately 11 times and over 7 times respectively higher in the GNB approaches than the SSB. After solid-liquid separation of manure, during liquid composting a change in aeration method from intermittent to continuous reduced the N and P loading about 50% and 47%, respectively. Although changing in solid composting method from turning only to turning+aeration improved the N removal efficiency by 30.5%, not much improvement in P removal efficiency was observed.

**Conclusion:**

Although the GNB approaches depict the impact of nutrients produced in the mixed crop-swine farms on the overall agricultural environment, the SSB approach shows the partitioning among different nutrient loss pathways and storage of nutrients within the soil system; thus, can help design sustainable nutrient management plans for the mixed crop-swine farms. The study also suggests that continuous aeration for liquid composting and turning+aeration for solid composting can reduce nutrient loading to the soil.

## INTRODUCTION

Combining crops with livestock at the farm level is known as a way of using nutrients more efficiently. Mixed crop-livestock farming practice increases food production by facilitating higher level of integration between crop and livestock production systems [1]. Intensification in mixed crop-livestock farming system increases agricultural production, but at a substantially higher environmental cost. Excessive nutrient inputs from livestock manure in intensive mixed crop-livestock farming systems often cause nutrient surpluses and lead to water and air pollution. A balance between inputs and outputs is therefore necessary to ensure both productivity and sustainability for intensively managed agroecosystems. Nutrient budgets have been used to understand nutrient cycling in agroecosystems as well as environmental indicators and regulatory policy instruments for nutrient management [2].

Nutrient budgets are developed based on the principle of ‘conservation of matter’ and widely used in environmental and socioeconomic studies. In many countries, nutrient budgets are incorporated as voluntary and mandatory regulations in nutrient management plans. Although nutrient budgets have been in practice for more than a century, well-documented and globally acceptable procedures are not available [2]. For instance, the European Union (EU) member countries use more than 50 different nutrient budgeting methods [3]. Considering the above, the current gross nutrient balance (GNB) approach developed by the Organisation for Economic Co-operation and Development has been acknowledged as a robust and reliable method for estimating nutrient budget by researchers and policymakers. However, the current GNB approach has some limitations and is not applicable in all circumstances [4]. Therefore, more detailed and need-based nutrient budgeting approaches are required to develop a sustainable nutrient management plan.

Along with economic development, agricultural practices in Korea have become more intensive and mixed crop-livestock farming systems are getting more popular. According to Statistics Korea, more than 82% of the livestock farms in Korea include croplands although the area is usually limited [5]. However, there are no nutrient management plan available for these mixed crop-livestock farms due to lack of proper nutrient budgeting approach. Rapid industrialization in swine farms is resulted in an increase of swine population from around 3.6 million in 1983 to 11.3 million in 2018 with a concomitant increase in manure production [5]. The Korean government has regulations such as the prohibition of direct use of livestock manure on agricultural land and stringent nutrient concentration limits in compost and effluents of livestock wastewater treatment process to reduce nutrient loading from livestock manures and protect the environment. The swine production and manure management system in Korea is quite different compared to other countries due to climatic conditions and the limited available arable land [6]. Swine manure in Korea is usually segregated into solid and liquid phases and composted prior to land application. Composting process (both solid and liquid) is generally carried out within the farms to reduce the environmental impact of land spreading of swine manure. Solid composting is done by aeration and turning+aeration (TA) method, while the liquid composting process is operated under continuous, intermittent, and no-aeration [6]. However, there is widespread speculation about the livestock sector in Korea that, nutrient loading from livestock manure is the main reason for water quality deterioration and soil nutrient surpluses. Nutrient budgets can therefore be used to evaluate both the environmental impact and sustainability of mixed crop-swine farming systems. Till now, most of the nutrient budgets in Korea were either developed at regional or watershed or field-scale from the biogeochemical perspective [7–9], but none of the studies reflected the real livestock manure treatment scenario in Korea. This present study was therefore conducted to ascertain a budgeting approach suitable for the mixed crop-swine farming system in Korea considering the manure treatment methods and suggest an effective manure treatment method to reduce on-farm nutrient loading to the soil.

## MATERIALS AND METHODS

### Nutrient budget methodology

In this study, we compared the current and ideal GNB approaches and the soil system budget (hereafter referred to as SSB) approach with particular reference to on-farm manure treatment processes to ascertain a suitable nutrient budgeting approach for the mixed crop-swine farms in Korea. The GNB (current and ideal) and SSB approaches are quite different in their system boundaries. The GNB approach considers extended soil surface as system boundary and includes nutrient losses from livestock barns and manure management systems to the air, water and soil environment, indicating overall environmental impact. The SSB approach provides detailed information on all the nutrient inputs, outputs including atmospheric (in the case of nitrogen [N]) and hydrologic (for both N and phosphorus [P]) loss of nutrients from the soil and identifies the major nutrient loss pathways. All the components and subcomponents of the nutrient budgets are shown in the Table 1 (a and b). Due to lack of data, components including inputs from crop residues, mineralization and outputs through fodder production and crop residues and immobilization were not considered in this study. The strengths and shortcomings of each budgeting approach were also discussed in brief.

Data obtained from farm census and lab estimation to gether with data collected from the published literature were used to construct the farm nutrient budgets [6,7,9–21]. Nutrient fluxes were used to convert the different components of the nutrient budgets from their original reported unit to common units (Table 2). Most of the fluxes used in this study were derived from the studies focused on Korea [6,7,9–14]. Seed nutrient inputs fluxes were obtained from Kremer [15]. No studies have reported N fluxes for denitrification from chemical fertilizer and compost in Korea, we therefore used denitrification fluxes for industrialized countries reported by Bouwman et al [16]. Due to lack of studies on N leaching losses from upland agricultural fields in Korea, leaching loss flux was derived from Takakai et al [21].

### Research design

This study was conducted in 2016 based on a set of three case study sites located in three different cities (Chuncheon, Hongcheon, Hwacheon) in Gangwon province, Korea. The multiple case study method was selected to represent a variety of mixed crop-swine farming systems within different contexts including agricultural practices and major swine manure treatment methods. Information on farm practices was collected through farm census using extensive questionnaires. Table 3 shows general information about the investigated farms. Information on manure management practices and agricultural characteristics of the selected farms are presented in Tables 4 and 5, respectively. Comparison of the different manure treatment methods was carried out to ascertain the most effective manure treatment method.

#### Quantifying nutrient inputs

##### i) Nutrient inputs from seeds

Data on seed nutrient inputs fluxes were collected from Kremer [15] and were calculated as:

(1) $${\text{N}}_{\text{seed}}={\text{Seed}}_{\text{N}}\times \text{A}$$

(2) $${\text{P}}_{\text{seed}}={\text{Seed}}_{\text{p}}\times \text{A}$$

where N_seed_ and P_seed_ are the nutrient inputs from seed and planting materials (kg/yr); Seed_N_ and Seed_P_ are the seed nutrient inputs fluxes (kg/ha/yr); and A is the agricultural land area (ha).

##### ii) Nutrient inputs from chemical fertilizer

Interviews with farm owners were conducted to collect information on chemical fertilizer application (FA) rates. Among the farms, farm 2 practices organic farming and therefore does not use any chemicals during crop production. Data on chemical FA rate and nutrient content were obtained from the RDA [17]. The nutrient inputs from chemical fertilizer were calculated using the following equations:

(3) $${\text{N}}_{\text{fert}}=\text{FA}\times {\text{C}}_{\text{N fert}}\times \text{A}$$

(4) $${\text{P}}_{\text{fert}}=\text{FA}\times {\text{C}}_{\text{P fert}}\times \text{A}$$

where N_fert_ and P_fert_ are the nutrient inputs from chemical fertilizer (kg/yr); FA is the fertilizer application rate (kg/ha/yr), C_N fert_ and C_P fert_ are the fertilizer nutrient content (%) and A is the agricultural land area for each crop (ha).

##### iii) Nutrient inputs from livestock manure

Data on using unit discharge fluxes of swine were obtained from the Ministry of Environment (MoE), Korea [10]. N and P content in swine manure were developed previously [6]. Information on bulking agents (BA) was collected during the farm census. N and P contents of the BA were obtained from the RDA [18]. The nutrient inputs from livestock manure were calculated using the following equations:

(5) $${\text{N}}_{\text{man}}=\text{AH}\times \text{D}\times {\text{C}}_{\text{N man}}+"\text{BA}\times {\text{C}}_{\text{N bed}}$$

(6) $${\text{P}}_{\text{man}}=\text{AH}\times \text{D}\times {\text{C}}_{\text{P man}}+\text{BA}\times {\text{C}}_{\text{P bed}}$$

where N_man_ and P_man_ are the nutrient inputs from livestock manure (kg/yr); AH is the number of animals (head); D is unit discharge coefficient (kg/head/yr); C_N man_ and C_P man_ are the nutrient content in swine manure (g/kg); BA is the bulking agent (kg) and C_N bed_ and C_P bed_ are the nutrient content in BA (g/kg).

##### iv) Nutrient inputs from compost

Swine farms in Korea segregate the swine manure into solid and liquid phases and following segregation, manure is composted [6]. Unit discharge fluxes for both solid (feces) and liquid (urine + wastewater) published by the MoE, Korea [10] were used to calculate individual farm manure production. Among the investigated farms, farm 1 does not have solid compost processing facility, whereas farm 2 and farm 3 have the both (solid and liquid) composting facilities. N and P content in swine manure (solid and liquid) and nutrient loading fluxes from solid and liquid manure were developed previously [6]. For ease of calculation, we assumed that nutrient produced in the farms are applied to the farm arable land. The following equations were used to estimate the N and P inputs from compost:

(7) $${\text{N}}_{\text{com}}={\text{N}}_{\text{sc}}+{\text{N}}_{\text{lc}}$$

(8) $${\text{P}}_{\text{com}}={\text{P}}_{\text{sc}}+{\text{P}}_{\text{lc}}$$

where N_com_ and P_com_ are the total nutrient inputs from compost (kg/yr); N_sc_ and N_lc_ are the N inputs from solid and liquid compost (kg/yr), respectively and P_sc_ and P_lc_ are the P inputs from solid and liquid compost (kg/yr), respectively. Nutrient inputs from solid and liquid compost were calculated separately using the equations below:

##### (i) Nutrient production from solid composts

(9) $${\text{N}}_{\text{sc}}=(\text{AH}\times {\text{D}}_{\text{s}}\times {\text{C}}_{\text{N}}\times {\text{LC}}_{\text{N}})+\text{BA}$$

(10) $${\text{P}}_{\text{sc}}=(\text{AH}\times {\text{D}}_{\text{s}}\times {\text{C}}_{\text{P}}\times {\text{LC}}_{\text{P}})+\text{BA}$$

where N_sc_ and P_sc_ are the nutrient inputs from solid compost (kg/yr); AH is the number of animals (head); D_s_ is the unit discharge coefficient for solid fraction (kg/head/yr); C_N_ and C_P_ are the nutrient content in solid fraction after separation (g/kg); LC_N_ and LC_P_ are nutrient loading fluxes and BA is the bulking agent (kg).

##### (ii) Inputs from liquid composts

(11) $${\text{N}}_{\text{lc}}=(\text{AH}\times {\text{D}}_{\text{l}}\times {\text{C}}_{\text{N}}\times {\text{LC}}_{\text{N}})$$

(12) $${\text{P}}_{\text{lc}}=(\text{AH}\times {\text{D}}_{\text{l}}\times {\text{C}}_{\text{P}}\times {\text{LC}}_{\text{P}})$$

where N_lc_ and P_lc_ are the nutrient inputs from liquid compost (kg/yr); AH is the number of animals (head); D_l_ is unit discharge coefficient for liquid fraction (kg/head/yr); C_N_ and C_P_ are the nutrient content in liquid fraction after separation (g/kg) and LC_N_ and LC_P_ are nutrient loading fluxes.

##### v) Nutrient inputs from atmospheric deposition

Values from previously published literature were used to estimate the N and P inputs from atmospheric deposition [9, 11] and expressed as:

(13) $${\text{N}}_{\text{atm}}={\text{AD}}_{\text{N}}\times \text{A}$$

(14) $${\text{P}}_{\text{atm}}={\text{AD}}_{\text{P}}\times \text{A}$$

where N_atm_ and P_atm_ are the nutrient inputs from atmospheric deposition (kg/yr); AD_N_ and AD_P_ are the unit atmospheric deposition fluxes (kg/ha/yr) and A is the agricultural land area (ha).

##### vi) Nitrogen input from biological nitrogen fixation

The amount of N input from biological nitrogen fixation (BNF) was calculated using standard values from the published literature [7] and the following equation was used for calculation:

(15) $${\text{N}}_{\text{bnf}}=\text{BNF}\times \text{A}$$

where N_bnf_ is the N input from BNF (kg/yr); BNF the unit biological nitrogen fixation fluxes (kg/ha/yr) and A is the agricultural land area (ha).

#### Quantifying nutrient outputs

##### i) Outputs in harvested crops

Data on crop production were collected during farm cen sus. N and P contents of the crops were obtained from the RDA [19]. The following equations were used to estimate nutrient outputs through crop harvesting:

(16) $${\text{N}}_{\text{crop}}={\text{Pro}}_{\text{crop}}\times {\text{C}}_{\text{N crop}}\times \text{A}$$

(17) $${\text{P}}_{\text{crop}}={\text{Pro}}_{\text{crop}}\times {\text{C}}_{\text{P crop}}\times \text{A}$$

where N_crop_ and P_crop_ are the nutrient outputs through crop harvesting (kg/yr); Pro_crop_ is the crop production (kg/ha/yr); C_N crop_ and C_P crop_ are the estimated crop nutrient content (%); A is the agricultural land area for each crop (ha).

##### ii) Changes in soil nutrient stocks

Data on soil nutrient stocks were obtained from RDA [20] and the following equations were used to estimate changes in soil nutrient stocks:

(18) $${\text{N}}_{\text{ssc}}=({\text{N}}_{\text{soil }2017}-{\text{N}}_{\text{soil }2016})\times \text{A}$$

(19) $${\text{P}}_{\text{ssc}}=({\text{P}}_{\text{soil }2017}-{\text{P}}_{\text{soil }2016})\times \text{A}$$

where N_ssc_ and P_ssc_ are the soil nutrient stock changes (kg/yr); N_soil 2017_ and P_soil 2017_ are the soil N and P content in the year 2017 (kg/ha/yr), respectively; N_soil 2016_ and P_soil 2016_ are the soil N and P content in the year 2016 (kg/ha/yr); A is the agricultural land area of the farm (ha).

##### iii) Output in atmospheric losses (volatilization and deni trification)

N loss through volatilization and denitrification were esti mated based on fluxes reported by Bouwman et al [16] and Bashkin et al [7] and calculated using the following formula:

##### (i) Volatilization loss

(20) $${\text{N}}_{\text{vol}}={\text{N}}_{\text{fert}}\times {\text{V}}_{\text{fert}}+{\text{N}}_{\text{com}}\times {\text{V}}_{\text{com}}$$

where N_vol_ is the nutrient output through volatilization (kg/yr); N_fert_ is the nutrient input from chemical fertilizer (kg/yr); V_fert_ is the volatilization coefficient of for applied chemical fertilizer (%); N_com_ is the nutrient input from compost (kg/yr); V_com_ is the volatilization coefficient for applied compost (%).

##### (ii) Denitrification loss

(21) $${\text{N}}_{\text{den}}={\text{N}}_{\text{fert}}\times {\text{D}}_{\text{fert}}+{\text{N}}_{\text{com}}\times {\text{D}}_{\text{com}}+\text{TA}\times {\text{D}}_{\text{agri}}$$

where N_den_ is the nutrient output through denitrification (kg/yr); N_fert_ is the nutrient input from chemical fertilizer (kg/yr); D_fert_ is the denitrification coefficient for applied chemical fertilizer (%); N_com_ is the nutrient input from compost (kg/yr); D_com_ is the denitrification coefficient for applied compost (%); TA is the total arable land area (ha); D_agri_ is the unit denitrification coefficient for arable land (kg/ha/yr).

##### iv) Outputs in hydrologic export (leaching and runoff)

Hydrologic export of nutrients was estimated in terms of leaching and runoff using fluxes derived from published literature [12–14,21] and expressed as:

##### (i) Nutrient export in leaching

(22) $${\text{N}}_{\text{leach}}={\text{N}}_{\text{input}}\times {\text{L}}_{\text{N}}$$

(23) $${\text{P}}_{\text{leach}}={\text{N}}_{\text{input}}\times {\text{L}}_{\text{P}}$$

where N_leach_ and P_leach_ are the nutrient outputs in leaching (kg/yr); N_input_ and P_input_ are the total nutrient inputs (kg/yr); L_N_ and L_P_ are the nutrient leaching fluxes (%).

##### (ii) Nutrient export in runoff

(24) $${\text{N}}_{\text{runoff}}={\text{N}}_{\text{input}}\times {\text{R}}_{\text{N}}$$

(25) $${\text{P}}_{\text{runoff}}={\text{N}}_{\text{input}}\times {\text{R}}_{\text{P}}$$

where N_runoff_ and P_runoff_ are the nutrient outputs in runoff (kg/yr); N_input_ and P_input_ are the total nutrient inputs (kg/yr); R_N_ and R_P_ are the nutrient runoff fluxes (%).

#### Nutrient balance

After quantifying all the nutrient inputs and outputs, the nutrient balance (surplus/deficit) was estimated using the following mass balance equations:

(26) N balance (surplus/deficit)=total inputs-total outputs

(27) P balance (surplus/deficit)=total inputs-total outputs

A positive nutrient balance indicates a nutrient surplus or potential for nutrient loss to the environment, while a negative nutrient balance signifies a nutrient deficit.

##### i) Atmospheric gross nitrogen surplus

The atmospheric gross nitrogen surplus (aGNS) was the sum of N loss before application in the soil and N loss after application from the soil and expressed as:

(28) $$\text{aGNs}={\text{N}}_{\text{NLB}}+{\text{N}}_{\text{NLA}}$$

where aGNS is the atmospheric gross nitrogen surplus (kg/yr); N_NLB_ is the N loss before application in the soil (kg/yr) and N_NLA_ is the N loss after application from the soil (kg/yr). N loss before application in the soil and after application from the soil were calculated separately using the equations below:

##### (i) Nitrogen loss before application in the soil

(29) $${\text{N}}_{\text{NLB}}={\text{N}}_{\text{man}}-{\text{N}}_{\text{com}}$$

where N_NLB_ is the N loss before application in the soil (kg/yr); N_man_ is the N input from livestock manure (kg/yr); N_com_ is the N input from compost (kg/yr).

##### (ii) Nitrogen loss after application in the soil

(30) $${\text{N}}_{\text{NLA}}={\text{N}}_{\text{vol}}+{\text{N}}_{\text{den}}$$

where N_NLA_ is the N loss after application in the soil (kg/yr); N_vol_ is the N output through volatilization (kg/yr); N_den_ is the N output through denitrification (kg/yr).

##### ii) Hydrologic gross nitrogen surplus

The hydrologic gross nitrogen surplus (hGNS) is estimated by deducting the aGNS from gross nitrogen surplus (GNS) and expressed as:

(31) hGNs=GNS-aGNS

## RESULTS AND DISCUSSION

### Nutrient budgets for individual farms

Based on Tables 1 (a, b), and 2, the N and P budgets for the mixed crop-swine farms were developed and are shown in Tables 6 and 7, respectively. Major sources of both nutrient inputs and outputs differ substantially according to the manure recycling processes and treatment methods used by the respective farms. The detailed explanations of nutrient budgets are described in the following subsections.

#### Nitrogen budgets

Table 6 shows the nitrogen budgets for the investigated farms using current and ideal GNB approaches and SSB approach. In both GNB approaches, all the components of N inputs are same except N inputs from livestock manure. Inputs from livestock manure were directly calculated by multiplying the livestock population with the excretion fluxes reported by MoE and were 11,075.9, 28,480.9, and 23,734.1 kg/yr for the farms 1, 2 and 3, respectively.

Whereas based on estimates, farms 1 and 2 produced 1,209.4 ×10^3^ and 3,084.7×10^3^ kg/yr of liquid compost, respectively using the intermittent aeration (IA) method, after separating solid fraction from the swine manure. Farm 3 produced 2,668.7 ×10^3^ kg/yr of liquid compost using the continuous aeration (CA) method. The N content in the liquid compost was estimated as 8 g/kg, while loading fluxes varied substantially depending on the manure treatment method. N loading fluxes for liquid composts produced by IA and CA were 0.52 and 0.26, respectively [6]. For solid composting, farm 2 used the TA method and produced 296×10^3^ kg/yr solid compost. On the other hand, farm 3 produced 273.39×10^3^ kg/yr solid compost using the turning (T) only method. The N content in the solid compost was 9.6 g/kg and loading fluxes for TA and T were estimated as 0.48 and 0.69, respectively [6]. Total N inputs from compost were calculated as 5,031.1, 14,196.4, and 7,362.1 kg/yr in farms 1, 2 and 3, respectively.

N inputs from chemical fertilizer and seeds used for plant ing crops added a relatively small amount of N to the farms due to limited arable land. Chemical fertilizer added only 8.0 and 16.0 kg/yr to farms 1 and 3, respectively. N inputs from the planting materials to farms 1, 2, and 3 were 0.3, 7.9, and 4.6 kg/yr, respectively. Other sources of N inputs to the farms were BNF and atmospheric deposition. In Korea, BNF for the dry field crops was estimated to be 15 kg/ha/yr [7] and amount of N fixed at the agricultural field in farms 1, 2 and 3 were 9.9, 49.5, and 34.8 kg/yr, respectively. Atmospheric deposition of N in Korea was estimated as 24.1 kg/ha/yr [11] and the amount of N inputs through atmospheric deposition were calculated as 15.9, 79.5, and 55.9 kg/yr farms 1, 2 and 3, respectively.

Although the GNB approach is generally regarded as a sound method of nutrient budget calculation considering the N outputs through crop harvesting and soil N stock changes; nutrient losses from soil were not taken into accounts. The SSB approach elucidated that most of the N was lost from the farms through leaching, followed by volatilization, runoff, denitrification and crop harvesting. N losses due to leaching were calculated using data from Takakai et al [21]. Leaching losses accounted for 26.5% of the total input and calculated as 1,342.3, 3,798.3, and 1,980.5 kg/yr for farm 1, 2 and 3, respectively. While runoff loss of N was reported as 15% of the total N input [12] and yielding an annual N output of 759.8, 2,150.0, 1,121.0 kg/yr from farms 1, 2 and 3, respectively. Atmospheric loss of N due to denitrification was calculated as 657.2, 1,855.4, and 966.5 kg/yr for farms 1, 2 and 3, respectively using the estimates reported by Bashkin et al [7]. Of the total chemical fertilizer and compost applied in the field, N loss through volatilization was estimated as 14% and 23%, respectively [16] and calculated as 1,158.3, 3,265.2, and 1,695.5 kg/yr for farms 1, 2 and 3, respectively. N export in crop harvesting was 26.4, 88.6, and 68.4 kg/yr for farms 1, 2 and 3, respectively.

#### Phosphorus budgets

P budgets for the selected farms using current and ideal GNB approaches and SSB approach are shown in Table 7. P inputs from swine manure were calculated as 1,954.6, 5,026.1, and 4,188.4 kg/yr. P levels in the liquid compost and solid compost were estimated as 0.4 and 3.6 g/kg, respectively and loading fluxes from liquid compost using IA and CA were 0.15 and 0.08, respectively [6]. Despite differences in treatment methods (TA and T) for solid composts, the loading fluxes did not vary much and reported as 0.94 and 0.96, respectively. Annual P inputs to farms 1, 2 and 3 were therefore calculated as 72.6, 1,208.0, and 1,010.6 kg/yr, respectively. Through chemical fertilizers, farms 1 and 3 received only 8.0 and 16.0 kg P/yr, respectively. Annual P inputs from seeds to the farms (1, 2, and 3) were calculated as 0.5, 3.0, and 2.0 kg/yr, respectively. Atmospheric deposition of P was estimated as 0.59 kg/ha/yr [9], which equivalent to 0.4, 1.9, and 1.4 kg/yr of P inputs to farm 1, 2 and 3, respectively.

The current GNB approach considered crop harvesting as the only pathway of P outputs, while in the Ideal GNB approach soil stock changes of P were also included. The SSB approach showed that most of the P left the farms through leaching, followed by crop harvesting and runoff. Of the total P applied, Kang et al [13] reported that 35.3% was lost due to leaching, corresponding to an amount of 28.8, 428.2, and 363.6 kg/yr for farms 1, 2 and 3, respectively. Runoff losses were calculated as 7.4% of the total input [14] and accounted for 6.0, 89.8, and 76.2 kg P/yr for farms 1, 2 and 3, respectively. P export in crop harvesting was 5.9, 19.2, and 14.9 kg/yr for the farm 1, 2 and 3, respectively.

### Trends in the nutrient budgets

N budgets for farms 1, 2, and 3 varied significantly among the budgeting approaches. The estimated total N inputs to the farms 1, 2, and 3 using the GNB approaches were approximately 11,110.0, 28,617.9, and 23,845.4 kg/yr, respectively. While estimated using the SSB approach, the total N inputs were 5,065.2, 14,333.4, and 7,473.4 kg/yr for the farms 1, 2, and 3. As expected, anthropogenic N inputs through livestock manure/compost were the largest source of N in the farms in all cases. Other sources including chemical fertilizers, planting materials, BNF and atmospheric deposition added only a small portion to the inputs. The total N outputs calculated using the current GNB approach were approximately 26.4, 88.6, and 68.4 kg/yr, the ideal GNB approach were 67.3, 146.1, and 21.9 kg/yr and the SSB approach were 3,943.9, 11,157.6, and 5,831.8 kg/yr for farms 1, 2 and 3, respectively. The SSB approach showed that hydrologic export (leaching and runoff) and atmospheric loss (volatilization and denitrification) were the two dominant pathways of N outputs, accounting for approximately 53.2% and 45.9% of the total N outputs (average), respectively. Crop harvesting accounted for only a small portion of N outputs (approximately 0.7%).

Similarly, a fairly large difference in the P budget using the GNB approaches and SSB approach was observed. Based on the GNB approaches, the total P inputs to farms 1, 2, and 3 were approximately 1,963.5, 5,031.0, and 4,207.8 kg/yr, respectively and SSB approach estimated the P inputs as 81.5, 1,212.9, and 1,030.0 kg/yr, respectively. P inputs from livestock manure/compost were the dominant source of nutrient in the farms. Other sources such as chemical fertilizers, planting materials and atmospheric deposition accounted for a relatively small amount of the total inputs. The total P outputs for farms 1, 2, and 3 using the current GNB approach were approximately 5.9, 19.2, and 14.9 kg/yr, respectively; the ideal GNB approach were −34.9, −27.3, and 219.3 kg/yr, respectively and the SSB approach were about 40.7, 537.1, and 454.7 kg/yr, respectively. The negative outputs found in the ideal GNB for the farms 1 and 2 implies that P removal from the soil P stocks. The SSB approach identified that P export in leaching was the major pathway of P outputs, accounting for about 76.7% (average) of the total P outputs. Of the total P outputs, on an average approximately 16.1% was lost due to runoff. Crop harvesting was accounted for 7.1% (average) of the total P outputs.

As expected, all the investigated farms showed nutrient surpluses for N and P. Nutrient surpluses in the farms are mainly attributed to livestock manure/compost application. Due to differences in system boundaries, a large variation in nutrient surpluses was observed among the budgeting approaches. In both GNB (current and ideal) approaches, nutrient inputs from livestock manure are considered, whereas as mentioned earlier livestock manure is composted before land application in Korea. Therefore, calculated nutrient budgets based on the GNB approaches ultimately showed large nutrient surpluses as the nutrient loss during composting were not taken under consideration while calculating the nutrient inputs. Moreover, the current GNB approach is unable to reflect the effects of different on-farm manure treatment methods on compost production and does not consider the nutrient loss pathways. In case of the ideal GNB approach, it can be used as a comprehensive agri-environmental indicator as it considers the aGNS to reflect the N loss and shows the potential risk of nutrient surplus to the whole agricultural environment (soil, air, and water). However, the system boundary in the ideal GNB is quite wide and hence requires many data for adequate calculation. Due to lack of data, its contribution to uncertainty is very high and thus difficult to use in managing the nutrients. Furthermore, in the ideal GNB approach, hGNS is not estimated directly rather calculated by deducting aGNS from the gross nutrient surplus. Depending on the manure treatment process, the aGNS varies so as hGNS. The ideal GNB approach therefore cannot depict real nutrient loss. Moreover, the manure management system in Korea is not only limited to solid-liquid separation and composting, but it also includes efforts towards reducing the odor using absorption tower, biofilter, biocurtain etc. So, not all the N lost through denitrification and volatilization are not entering the atmosphere, a portion of N is captured by the odor reduction facilities. So, there is always a chance of overestimating the aGNS in the ideal GNB approach. Despite a detailed approach of calculating nutrient balance, the ideal GNB approach therefore cannot depict the real N loss. The same is true for P also. Although unlike N, P cannot be lost during the manure treatment and composting atmospherically; rather it reacts with other cations and precipitates as struvite. Struvite either deposits on the digester or form scales in the pipes during the closed manure treatment system. In both GNB approaches, P removal through struvite precipitation and loss due to scale formation are not considered. Whereas the SSB approach focuses only on the soil environment. It is a nutrient budgeting approach providing sufficient information on nutrient inputs and outputs, nutrient recycling process and dominant nutrient loss pathways. The SSB approach considers the amount of nutrient applied to the soil after manure treatment as solid and liquid compost. Thereby the difference in the nutrient inputs by the different manure recycling and treatment processes method can be considered directly. Although the nutrient loss to the atmosphere before the application is not considered in the SSB approach, the dominant nutrient loss pathways from after application can easily be identified. The SSB approach also shows the partitioning among different nutrient loss pathways and storage or depletion of nutrients within the soil system. From the above discussion, it can be said that, although the ideal GNB is a detailed nutrient budgeting approach depicting the impact of nutrients produced in the mixed crop swine farms on overall agricultural environment, the SSB approach appears to be a reasonable budgeting approach suitable for the mixed crop-swine farms in Korea and more appropriate for the farmers for efficient nutrient management.

### Effect of manure treatment methods on nutrient loading and budgets

Nutrient inputs in agroecosystems above requirements result in nutrient surplus and are one of the main causes of eutrophication in freshwater ecosystems. Intensive livestock farming systems without appropriate manure recycling processes and treatment methods generate large nutrient surpluses [22]. In this study, the SSB approach revealed that on an average approximately 22.1% of applied N and 53.9% of the applied P was retained in the farms as surpluses and compost was the dominant source of nutrient inputs. Hence, we quantified and compared N and P produced from swine manure through different recycling processes and treatment methods using nutrient loading fluxes reported by Won et al [6] to suggest an effective on-farm treatment method for reduction of nutrient loading to soil (Table 8).

For liquid composting, changing to the aeration method from intermittent to continuous reduced the N and P loading about 50% and 47%, respectively. These findings are supported by previous studies [23]. Factors such as influent characteristics, pH, molar ratio of NH_4_, PO_4_, and Mg, aeration and temperature control nutrient removal from swine wastewater [24]. Among the above-mentioned factors, aeration plays a significant role in nutrient removal. Continuous aeration influences the wastewater pH by CO_2_ stripping and thus improves the removal efficiency of nutrients [25]. As CA during liquid composting increases pH, struvite can be formed in alkaline conditions. Struvite produced in the digester either precipitates or forms scale in the piping system as described above. Such struvite cannot be applied in the arable lands but plays an important role in removing nutrients from the liquid manure. Based on the removal efficiency, the CA method during liquid composting is suggested.

Turning (T) only and TA, are the two methods used by the swine farms in Korea for solid composting. Table 5 shows that change in treatment method from T to TA improved the N removal efficiency by 30.5%, while P removal efficiency decreased by 2.1%. A similar phenomenon was reported by Zhang and He [26]. Due to microbial nitrification and atmospheric loss through denitrification and ammonia volatilization, N concentration decreased in the solid compost using the TA method [27]. Unlike N, P is less mobile in the environment and less susceptible to loss during the composting process. Therefore, the TA method of solid composting resulted in decreased N loading, while P loading remained almost the same. Considering the decrease in N loading we therefore support the use of the TA method of solid composting over the T method.

Furthermore, the impacts of suggested treatment methods on nutrient budgets were evaluated by developing soil system N and P budgets for the study farms (CA for liquid composting and TA for solid composting) (Tables 9, 10). The results showed that changing swine manure treatment methods significantly reduced the farm nutrient surpluses. For farm 1, change in manure treatment method resulted in 49.4% and 47.6% reduction in N and P surplus, respectively. While for farm 2, 44.6% and 7.3% reduction in surplus was observed for N and P, respectively. For farm 3, although the N surplus decreased by 7.4%, the P surplus increased by 1.9%.

### Management of surplus nutrients

Effective management of surplus nutrients while achieving agro-economic sustainability and crop productivity poses many challenges. Due to rapid industrialization and urbanization, arable land in Korea is decreasing. Like other countries, farmers in Korea are therefore shifting towards high fertilizer (chemical and compost) input-based farming systems to ensure productivity. Farmers generally think that the more fertilizer they use, higher yield and profit will result. Intensive agricultural practices in Korea started during the mid-1980s and increased until the mid-1990s. Due to the development of national soil fertility database, decision-support system for farmers and promotion of eco-friendly agricultural policies, FA decreased from 689,901 metric tons in 2002 to 450,453 metric tons in 2016 [5]. However, the numbers are still higher than the most OCED member states. Recent N and P budget studies reported that FA rate for highland crops in Korea is more than 1.5 and 4.5 times, respectively higher than the recommended rate [8,9]. Such activities resulted in the retention of surplus nutrients on arable land. Hence, while managing the surplus nutrients along with the environmental aspects, the social and economic perspective should be considered. The fertilizer (chemical and compost) application rate to the crop fields often determined based on crop N requirement [28]. As a result, P may be applied above the crop P demand and retained in the soil. The retained P can be released in the future due to hydrologic, geologic, climatic events or changes in land management practices and can amplify P related environmental problems.

Moreover, the buildup of large nutrient surpluses from nutrients in animal feeds is another major issue in countries with intensive livestock production like Korea. Goulding et al [29] reported that low conversion efficiency of nutrients in animal feeds lead to loss of nutrient through excretion. For efficient nutrient management in Korea, swine farms either need to treat the manure using on-farm treatment facilities or send the wastewater to the centralized treatment plants before discharge into water bodies [30]. The operational cost of the farms having on-farm manure treatment processes is higher than the farms having a simple storage facility. Considering the reduction in nutrient content, post-management cost, social cost and environmental benefits, composting is an effective way to treat the manure. The farms already having composting facilities required no further investment in infrastructure development except installing the aeration system for solid composting. However, operational cost of liquid and solid composting by CA and TA, respectively might be higher than IA and turning. Acknowledging the environmental sustainability and long-term benefits, the Korean government could develop policy and provide subsidies to encourage the farm owners to change the on-farm treatment facilities. Therefore, improving on-farm manure treatment methods along with additional policies (tested and successful in other parts of the world) such as nutrient recovery from wastewater, reutilizing recovered nutrients as fertilizer and feed additives, extensive and organic farming systems, cultivating high yielding varieties for efficient use nutrients, precision farming, distributing compost in nearby specialized croplands, restricting maximum application limit and time, incentives for achieving balance and comprehensive nutrient management plans could be useful in reducing the nutrient surpluses in mixed crop-swine farms [29].

## IMPLICATIONS

The soil system budgeting approach considers all the possible nutrient inputs and identifies the dominant nutrient loss pathways and can therefore be considered as a rational budgeting approach for the mixed crop-swine farms. Combination of continuous aeration for liquid composting and turning+ aeration for solid composting can reduce the nutrient loading to the soil and nutrient surpluses. The findings of this research have several important implications for nutrient management for future practice. However, sensitivity analysis of the fluxes used in this study, economic analysis of the suggested on-farm manure treatment methods and more field studies are required for better understanding and reliability of the soil system budget approach in the Korean context.

## Figures and Tables

**Table 1:** Components of current and ideal GNB approaches and SSB approach to nutrient budgeting in mixed crop-swine farms used in this study

Current GNB of OECD	Ideal GNB of OECD	SSB
(a) N budget		
Input		
IN_N1_) Chemical fertilizer	IN_N1_) Chemical fertilizer	IN_N1_) Chemical fertilizer
IN_N2_) Livestock manure	IN_N2_) Livestock manure	IN_N2′_) Compost (solid and liquid)
IN_N3_) Net manure import/export, withdrawals, stocks	IN_N3_) Net manure import/export, withdrawals, stocks	IN_N3_) Organic fertilizer1)
IN_N4_) Organic fertilizer1)	IN_N4_) Organic fertilizer1)	IN_N4_) Biological N fixation
IN_N5_) Biological N fixation	IN_N5_) Biological N fixation	IN_N5_) Atmospheric deposition
IN_N6_) Atmospheric deposition	IN_N6_) Atmospheric deposition	IN_N6_) Mineralization
IN_N7_) Planting materials	IN_N7_) Planting materials	IN_N7_) Planting materials
	IN_N8_) Crop residues	
Total inputs = Sum (IN _N1_,IN_N2_,IN_N3_,IN_N4_,IN_N5_,IN_N6_,IN_N7_)	Total inputs = Sum (IN _N1_,IN_N2_,IN_N3_,IN_N4_,IN_N5_,IN_N6_,IN_N7_,IN_N8_)	Total inputs = Sum (IN_N1_,IN_N2_,IN_N3_,IN_N4_,IN_N5_,IN_N6_,IN_N7_)
Output		
OUT_N1_) Crop production	OUT_N1_) Crop production	OUT_N1_) Crop production
OUT_N2_) Fodder production2)	OUT_N2_) Fodder production2)	OUT_N2_) NH_3_ volatilization
OUT_N3_) Crop residues	OUT_N3_) Crop residues	OUT_N3_) Denitrification
	OUT_N4_) Stock changes of N in soil	OUT_N4_) Leaching
		OUT_N5_) Runoff
		OUT_N6_) Immobilization
Total outputs = Sum (OUT _N1_,OUT_N2_,OUT_N3_)	Total outputs = Sum (OUT _N1_,OUT_N2_,OUT_N3_,OUT_N4_)	Total outputs = Sum (OUT _N1_,OUT_N2_,OUT_N3_,OUT_N4_,OUT_N5_, OUT_N6_)
Surplus		
SP_N1_) Gross N surplus (input-output)	SP_N1_) Gross N surplus (input-output)	SP_N1_) N surplus (input-output)
	SP_N2_) Atmospheric gross nitrogen surplus	
	SP_N2-1_) N loss before application in the soil	
	SP_N2-2_) N loss after application soil	
	SP_N3_) Hydrologic gross N surplus	
	(SP_N1_-SP_N2_)	
(b) P budget		
Input		
IN_P1_) Chemical fertilizer	IN_P1_) Chemical fertilizer	IN_P1_) Chemical fertilizer
IN_P2_) Livestock manure	IN_P2_) Livestock manure	IN_P2′_) Compost (solid and liquid)
IN_P3_) Net manure import/export, withdrawals, stocks	IN_P3_) Net manure import/export, withdrawals, stocks	IN_P3_) Organic fertilizer1)
IN_P4_) Organic fertilizer1)	IN_P4_) Organic fertilizer1)	IN_P4_) Atmospheric deposition
IN_P5_) Atmospheric deposition	IN_P5_) Atmospheric deposition	IN_P5_) Mineralization
IN_P6_) Planting materials	IN_P6_) Planting materials	IN_P6_) Planting materials
	IN_P7_) Crop residues	
Total inputs = Sum (IN _P1_,IN_P2_,IN_P3_,IN_P4_,IN_P5_,IN_P6_)	Total inputs = Sum (IN _P1_,IN_P2_,IN_P3_,IN_P4_,IN_P5_,IN_P6_,IN_P7_)	Total inputs = Sum (IN _P1_,IN_P2_,IN_P3_,IN_P4_,IN_P5_,IN_P6_)
Output		
OUT_P1_) Crop production	OUT_P1_) Crop production	OUT_P1_) Crop production
OUT_P2_) Fodder production2)	OUT_P2_) Fodder production2)	OUT_P2_) Leaching
OUT_P3_) Crop residues	OUT_N3_) Crop residues	OUT_P3_) Runoff
	OUT_P4_) Stock changes of P in soil	
Total outputs = Sum (OUT _P1_,OUT_P2_,OUT_P3_)	Total outputs = Sum (OUT _P1_,OUT_P2_,OUT_P3_,OUT_P4_)	Total outputs = Sum (OUT _P1_,OUT_P2_,OUT_P3_)
Surplus		
SP_P1_) P surplus (input-output)	SP_P1_) P surplus (input-output)	SP_P1_) P surplus (input-output)

GNB, Gross Nutrient Balance; OECD, Organisation for Economic Co-operation and Development; SSB, Soil System Budget; P, Phosphorus.

1) Not used in the farms.

2) Not produced in the farms.

**Table 2:** Values of nutrient fluxes from the literature used to calculate nutrient budgets in this study

Variables		Flux	References
N budget			
Seed N inputs	Sesame	0.4 kg/ha/yr	[15]
	Corn	4.4 kg/ha/yr	
Biological N fixation		15.0 kg/ha/yr	[7]
Atmospheric deposition		24.1 kg/ha/yr	[11]
Atmospheric loss			
Denitrification			[7]
Chemical fertilizer		15.0%	
Compost		13.0%	
Arable land		3.0 kg/ha/yr	
Volatilization			[16]
Chemical fertilizer		14.0%	
Compost		23.0%	
Hydrologic loss			
Leaching		26.5%	[21]
Runoff		15.0%	[12]
P budget			
Seed N inputs	Sesame	0.7 kg/ha/yr	[15]
	Corn	1.1 kg/ha/yr	
Atmospheric deposition		0.59 kg/ha/yr	[9]
Hydrologic loss			
Leaching		35.3%	[13]
Runoff		7.4%	[14]

N, nitrogen; P, phosphorus.

**Table 3:** Descriptive information for the farms investigated in this study

Contents	Farm 1	Farm 2	Farm 3
Location	Chuncheon	Hongcheon	Hwacheon
Latitude	37.8813°N	37.6970°N	38.1061°N
Longitude	127.7300°E	127.8887°E	127.7067°E
Altitude (masl)1)	99	252	109
Rainfall (mm/yr)	1,351	1,162	1,350
Mean annual temperature (°C)	11.4	11.2	11.4
No. of animal (head)	700	1,800	1,500
Excretion rate (kg/head/d)		5.1	
Manure production (×10^3^ kg/yr)	1,303	3,350	2,792
Bulking agent	-	Rice hull	Rice hull
Bulking agent usage (×10^3^ kg/yr)	-	16.7	13.9

1) Meters above sea level.

**Table 4:** Information on manure management of the farms investigated in this study

Manure handling practices and production			
Manure collection system	Slurry	Slurry	Slurry
Moisture content of solid after separation (%)	-	85	85
Recycle process and treatment method	Liquid composting with intermittent aeration	Solid composting with turning and aeration; liquid composting with intermittent aeration	Solid composting with turning; liquid composting with continuous aeration
Liquid compost			
No. of composting tank	1	1	3
Tank capacity (m^3^)	200	200	200
Surface area (m^2^)	50	50	50
Production amount (×10^3^ kg/yr)	1,209.4	3,084.7	2,668.9
Solid compost			
Production amount (×10^3^ kg/yr)	-	296	273.4

**Table 5:** Agricultural characteristics of the farms investigated in this study

Items	Farm 1	Farm 2		Farm 3	
Cultivated crop (s)	Sesame	Corn	Sesame	Corn	Sesame
Arable land (ha)	0.66	1.65	1.65	0.93	1.39
Chemical fertilizer (kg/ha/yr)	100	-	-	120	80
Growing season per yr			Single		
Production (kg/ha)	500	5,030	1,200	5,030	1,200

**Table 6:** N budgets for the mixed crop-swine farm systems investigated in this study

Items	Amount (kg N/yr)								

	Farm-1			Farm-2			Farm-3		
		
	Current GNB	Ideal GNB	SSB	Current GNB	Ideal GNB	SSB	Current GNB	Ideal GNB	SSB
Chemical fertilizer	8.0	8.0	8.0	-	-	-	16.0	16.0	16.0
Livestock manure	11,075.9	11,075.9		28,480.9	28,480.9	-	23,734.1	23,734.1	-
Compost	-	-	-	-	-	-	-	-	-
Liquid compost	-	-	5,031.1	-	-	12,832.6	-	-	5,551.2
Solid compost	-	-		-	-	1,363.8	-	-	1,810.9
Biological N fixation	9.9	9.9	9.9	49.5	49.5	49.5	34.8	34.8	34.8
Atmospheric deposition	15.9	15.9	15.9	79.5	79.5	79.5	55.9	55.9	55.9
Planting materials	0.3	0.3	0.3	7.9	7.9	7.9	4.6	4.6	4.6
Total inputs	11,110.0	11,110.0	5,065.2	28,617.9	28,617.9	14,333.4	23,845.4	23,845.4	7,473.4
Crop harvesting	26.4	26.4	26.4	88.6	88.6	88.6	68.4	68.4	68.4
Stock changes of N in soil	-	40.9	-	-	57.5	-	-	−46.5	-
Denitrification	-	-	-	-	-	-	-	-	-
Chemical fertilizer	-	-	1.2	-	-	0.0	-	-	2.4
Compost	-	-	654.0	-	-	1,845.5	-	-	957.1
Arable land	-	-	2.0	-	-	9.9	-	-	7.0
Volatilization	-	-	-	-	-	-	-	-	-
Chemical fertilizer	-	-	1.1	-	-	0.0	-	-	2.2
Compost	-	-	1,157.2	-	-	3,265.2	-	-	1,693.3
Leaching	-	-	1,342.3	-	-	3,798.3	-	-	1,980.5
Runoff	-	-	759.8	-	-	2,150.0	-	-	1,121.0
Total outputs	26.4	67.3	3,943.9	88.6	146.1	11,157.6	68.4	21.9	5,831.8
Gross N surplus	11,083.6	11,042.7	1,121.2	28,529.2	28,471.7	3,175.8	23,777.1	23,823.6	1,641.6
Atmospheric GNS	-	6,044.8	-	-	14,284.5	-	-	16,372.0	-
Hydrologic GNS	-	4,997.9	-	-	14,187.2	-	-	7,451.6	-

GNB, gross nutrient balance; SSB, soil system budget; N, nitrogen; GNS, gross nitrogen surplus.

**Table 7:** P budgets for the mixed crop-swine farm systems investigated in this study

Items	Amount (kg P/yr)								

	Farm-1			Farm-2			Farm-3		
		
	Current GNB	Ideal GNB	SSB	Current GNB	Ideal GNB	SSB	Current GNB	Ideal GNB	SSB
Chemical fertilizer	8.0	8.0	8.0	-	-	-	16.0	16.0	16.0
Livestock manure	1,954.6	1,954.6	-	5,026.1	5,026.1	-	4,188.4	4,188.4	-
Compost	-	-	-	-	-	-	-	-	-
Liquid compost	-	-	72.6	-	-	185.1	-	-	85.4
Solid compost	-	-	-	-	-	1,022.9	-	-	925.2
Atmospheric deposition	0.4	0.4	0.4	1.9	1.9	1.9	1.4	1.4	1.4
Planting materials	0.5	0.5	0.5	3.0	3.0	3.0	2.0	2.0	2.0
Total inputs	1,963.5	1,963.5	81.5	5,031.0	5,031.0	1,212.9	4,207.8	4,207.8	1,030.0
Crop harvesting	5.9	5.9	5.9	19.2	19.2	19.2	14.9	14.9	14.9
Stock changes of P in soil	-	−40.8	-	-	−46.5	-	-	204.4	-
Leaching	-	-	28.8	-	-	428.2	-	-	363.6
Runoff	-	-	6.0	-	-	89.8	-	-	76.2
Total outputs	5.9	−34.9	40.7	19.2	−27.3	537.1	14.9	219.3	454.7
P surplus	1,957.6	1,998.4	40.8	5,011.8	5,058.3	675.8	4,192.8	3,988.4	575.2

GNB, gross nutrient balance; SSB, soil system budget; P, phosphorus.

**Table 8:** Nutrient loading of different on-farm manure treatment methods for the study farms

Farm	Recycle processes and treatment methods		Nutrient loading (kg/yr)	

			N	P
Farm 1	Liquid composting	Intermittent aeration1)	5,031.1	72.6
		Continuous aeration	2,515.6	38.7
		Storage only	6,482.4	440.2
Farm 2	Solid composting	Turning and aeration1)	1,363.8	1,022.9
		Turning	1,960.7	1,001.7
	Liquid composting	Intermittent aeration1)	12,832.6	185.1
		Continuous aeration	6,416.2	98.7
		Storage only	16,533.9	1,122.8
Farm 3	Solid composting	Turning and aeration	1,259.8	944.8
		Turning1)	1,810.9	925.2
	Liquid composting	Intermittent aeration	11,102.5	160.1
		Continuous aeration1)	5,551.2	85.4
		Storage only	14,305.1	971.5

N, nitrogen; P, phosphorus.

1) Treatment methods used by the farms.

**Table 9:** Soil N budgets for the mixed crop-swine farm systems using suggested manure treatment methods in this study

Items	Amount (kg N/yr)		

	Farm-1	Farm-2	Farm-3
N inputs			
Planting materials	0.3	7.9	4.6
Chemical fertilizer	8.0	-	16.0
Compost			
Liquid compost	2,515.6	6,416.2	5,551.2
Solid compost	-	1,363.9	1,259.8
Biological N fixation	9.9	49.5	34.8
Atmospheric deposition	15.9	79.5	55.8
Total inputs	2,549.43	7,909.99	6,918.28
N outputs			
Crop harvesting	26.4	88.6	68.4
Denitrification			
Chemical fertilizer	1.2		2.4
Compost	327.0	1011.4	885.4
Arable land	1.9	9.9	7.9
Volatilization			
Chemical fertilizer	1.2	-	2.2
Compost	578.6	1,789.4	1,566.5
Leaching	675.6	2,096.1	1,833.3
Runoff	382.4	1,186.5	1,037.7
Total outputs	1,994.3	6,181.9	5,403.9
Balance (surplus)	555.1	1,728.0	1,514.3

N, nitrogen.

**Table 10:** Soil P budgets for the mixed crop-swine farm systems using suggested manure treatment methods in this study

Items	Amount (kg P/yr)		

	Farm-1	Farm-2	Farm-3
P inputs			
Planting materials	0.5	3.0	2.0
Chemical fertilizer	8.0	-	16.0
Compost			
Liquid compost	38.7	98.7	85.4
Solid compost	-	1,023.0	944.8
Atmospheric deposition	0.4	1.9	1.4
Total inputs	47.6	1,126.6	1,049.6
P outputs			
Crop harvesting	5.90	19.20	14.90
Leaching	16.78	397.25	370.09
Runoff	3.53	83.48	77.78
Total outputs	26.21	499.94	462.77
Balance (surplus)	21.38	626.70	586.84

P, phosphorus.
